# Trump and mRNA technologies: global health risks and opportunities in vaccine manufacturing diversification

**DOI:** 10.1590/0102-311XEN052426

**Published:** 2026-10-12

**Authors:** Henrique Zeferino de Menezes, Kenneth C. Shadlen

**Affiliations:** 1 Universidade Federal da Paraíba, João Pessoa, Brasil.; 2 London School of Economics and Political Science, London, U.K.

## Background: U.S. disengagement with mRNA technologies

On the first day of his second term, Donald Trump announced that the United States would be leaving the World Health Organization (WHO). One year later, on January 22, an official statement announced the conclusion of the process and the formal termination of U.S. membership in the organization ^1^. This decision triggered significant direct effects on global health governance, most notably the loss of the organization’s largest contributor and the disruption of the U.S.-WHO technical collaboration ^2^.

The WHO plays a central role in global disease surveillance, early warning systems, and the coordination of international responses to public health emergencies. After the COVID-19 pandemic, it assumed a strategic role in pharmaceutical technology cooperation and in promoting equitable access to health technologies. In 2021, the WHO launched the mRNA Technology Transfer Program to build sustainable regional capacity for the development and production of mRNA-based technologies in low- and middle-income countries ^3^ ^,^ ^4^.

Although the United States did not directly fund the program, its withdrawal from the WHO and the substantial cut in the WHO’s projected budget create challenges for the program’s continuity and expansion. The WHO recently announced the program’s second phase (2026-2030), designed to take a product-oriented approach by scaling up the commercial production of mRNA-based vaccines and therapeutics among participating firms ^5^.

The Trump administration has also adopted measures that weaken the United States science and innovation system, with direct effects on pharmaceutical innovation ^6^ ^,^ ^7^. A particularly controversial decision was the withdrawal of federal support for mRNA vaccine development under the Biomedical Advanced Research and Development Authority (BARDA). The measure canceled research grant contracts totaling approximately USD 500 million, affecting ongoing mRNA projects involving universities, research institutes, and major pharmaceutical companies. It also stipulated that no new mRNA-based projects would be initiated or funded in the future ^8^.

The situation worsened when the U.S. Food and Drug Administration (FDA) made the rare decision to refuse to review Moderna’s application for the first mRNA-based influenza vaccine ^9^, following public criticism by United States authorities of mRNA technology and despite the agency’s earlier approval of the company’s Phase 3 study design ^10^. The decision was reversed only after substantial criticism and pressure ^11^. This episode, together with other government actions that have weakened vaccine and drug authorization procedures and undermined national vaccination policy ^12^, has drawn criticism from leading U.S. pharmaceutical firms ^13^. Combined, these measures are likely to increase regulatory uncertainty and raise investor concerns, putting existing pharmaceutical investments at risk and potentially discouraging further investment in research and development ^14^.

## mRNA: beyond pandemic preparedness

The withdrawal of U.S. government support and regulatory uncertainties may lead to a severe U.S. disengagement from mRNA technology, with far-reaching global implications. Despite logistical challenges related to transport, storage, and deployment ^15^, mRNA-based vaccines are particularly well suited to respond to future epidemics involving novel pathogens, re-emerging threats, and viral variants ^16^. Their effectiveness lies in the ability to rapidly design and update vaccines, combined with the platform’s flexibility and scalable manufacturing capacity, which enable large-scale production within short time frames ^17^.

This technology also holds significant potential for vaccines against diseases for which there is no existing immunization. Beyond COVID-19, mRNA is expected to have broad applicability across viruses, bacteria, fungi, and parasites, as illustrated by the recent authorization of an mRNA vaccine against respiratory syncytial virus (RSV). In this context, there is considerable optimism about the development of flu vaccine candidates, given the adaptability of mRNA platforms. Moreover, mRNA-based approaches are expected to advance the treatment of noncommunicable and complex diseases ^18^. This versatility is expected to position mRNA as a platform for future biomedical innovation, enabling next-generation vaccines and therapeutic interventions across diverse health challenges.

## A global call for diversification of vaccine development and production

The challenges resulting from the U.S. defunding of the WHO and the U.S. withdrawal from mRNA initiatives weaken global preparedness for future pandemics and may delay the development of life-saving vaccines and therapeutics, underscoring the critical importance of geographically diversifying vaccine development and expanding manufacturing capacity for mRNA vaccines ^19^.

The COVID-19 pandemic showed that expanding sustainable vaccine production capacity in developing countries is essential for pandemic preparedness, ensuring timely access to vaccines and helping to bring the pandemic under control ^20^. The WHO Pandemic Agreement, adopted at the 78th World Health Assembly in 2025, reinforces this approach, including legally binding commitments to promote technology transfer to build a global network of producers, especially in developing countries, capable of rapid manufacturing of pandemic-related health products ^21^ ^,^ ^22^. However, the geographic diversification of pharmaceutical development and production capacity has effects beyond pandemic response. It also helps reduce inequalities in access to basic and routine vaccines by expanding manufacturing capacity in developing countries, increasing availability, and lowering prices ^23^ ^,^ ^24^. Strengthening research and development capacity in these countries also enables the development of new vaccines for neglected diseases and the improvement of existing vaccines, thereby improving vaccination coverage and equity ^25^.

## South America as a strategic hub

Considering the social and political crises triggered by the COVID-19 pandemic and the growing global demand for health equity, developing countries are advancing initiatives to strengthen local pharmaceutical innovation and manufacturing. These efforts include strategic coalitions between high- and middle-income countries (e.g., the G20 Coalition for Local and Regional Production, Innovation, and Equitable Access), cooperation among developing countries (e.g., the BRICS Vaccine Center), and regional initiatives such as the Partnership for African Vaccine Manufacturing (PAVM). These countries are also significantly engaged with the WHO mRNA Technology Transfer Program. Beyond the original hub in South Africa, 14 additional countries are now participating, each at different stages of developing mRNA capacity ^26^.

In this context, South America represents a strategic space for the development of mRNA-based technologies. A landmark initiative is underway in Argentina, where Sinergium Biotech is leading the development of an mRNA-based candidate vaccine against avian influenza (H5N1), in partnership with the Pan American Health Organization (PAHO) and with support from the Coalition for Epidemic Preparedness Innovations (CEPI). The project, already in preclinical trials, aims to establish proof of concept to enable technology sharing with other partners, strengthening regional preparedness for future pandemics. In parallel, Sinergium is advancing the construction of an mRNA vaccine production facility, scheduled to begin operations in 2026 ^27^.

Brazil is also advancing its technological capacities for mRNA-based technologies as part of a broader national strategy to develop a stronger health-industrial complex, aiming to strengthen domestic production and innovation in vaccines and other health technologies. Oswaldo Cruz Foundation (Fiocruz, acronym in Portuguese), also selected as part of the WHO mRNA Technology Transfer Program, has advanced in building technological capacities, achieving concrete results, such as the first national mRNA platform, with a filed patent application ^28^. Brazil has also been strengthening cooperation within BRICS and the G20 to advance the development of an mRNA-based tuberculosis vaccine ^29^.

These cases illustrate the potential of geographically diversifying vaccine production capacity not only to increase the resilience of the regional innovation system, but also to expand global capacity for vaccine development, strengthen pandemic preparedness, and enable the development of new vaccines for diseases that affect large populations and for which vaccine supply may still be insufficient. Human avian influenza, for example, still lacks a licensed human vaccine, despite being an emerging infectious disease with high pandemic potential and significant public health risk. Meanwhile, the development of a new tuberculosis vaccine could help improve the control of a disease that still affects over 10 million people annually and causes more than 1 million deaths each year, particularly in developing countries.

## The future of mRNA in South America

The success of policies aimed at developing and producing vaccines and other complex health technologies depends on strong coordination among governments, firms, and international partners. These actors must ensure adequate funding for research and development, invest in scientific capacity and workforce training, build and maintain appropriate infrastructure, and mitigate financial risks.

Argentina and Brazil can help position South America as a strategic hub for vaccine research and development. The experiences of both countries during the COVID-19 pandemic, when they engaged in technology transfer agreements to support local vaccine production, provide important lessons ^30^. In addition, both countries possess infrastructure, technical expertise, and scientific capabilities that can support further advances in the development and production of mRNA vaccines.

However, this process faces barriers. Argentina’s recent withdrawal from the WHO may limit its participation in international health cooperation networks and reduce the opportunities for technical collaboration available to its pharmaceutical companies. Another structural challenge to sustaining vaccine production in the region is ensuring demand for newly developed and regionally produced vaccines. This challenge can be mitigated by strengthening and strategically coordinating public purchasing power beyond individual national public health systems. Even greater benefits could be achieved through stronger political and technical coordination at the regional level. The use of pooled procurement agreements, leveraging existing regional cooperation mechanisms such as Mercosur and the PAHO Revolving Fund, could help ensure demand sustainability and mitigate financial risks.

The recent experience of the COVID-19 pandemic and the actions taken by the U.S. highlight the critical importance of geographically diversifying vaccine development and production. These events show that global health security depends on building resilient and distributed capacities for mRNA and other advanced health technologies. South America, through initiatives in Argentina and Brazil and with the support of the WHO and PAHO, could emerge as a strategic hub for mRNA technologies, strengthening local innovation, boosting regional preparedness for future pandemics, and supporting equitable access to advanced health technologies. Achieving this potential requires sustained national political commitment, as well as stronger political and technical coordination at the international and regional levels.

## Data Availability

Not applicable.
